# A Case of Cyanosis

**Published:** 1850-06

**Authors:** William Waters

**Affiliations:** Fredericktown, Maryland


*A Case of Cyanosis.* By William Waters, M. D., Fredericktown, Maryland.

Mr. Wm. R. Y., of small stature and delicate frame, aged forty years, within seventeen days. According to the history given by his mother, Mr. Y. gave no evidence of morbus cœruleus until he was about six years of age ; the seizure at this time was ascribed by her to an attack of measles, although the paroxysm came on after his recovery, and followed exercise in running about the yard. From that period until his death, he was subject to paroxysms on exercise, mental emotions, and occasionally after eating a full meal. The lividity affected his lips, face, hands and arms. When slight, the lips only would be affected, and the shade would vary from a partial blue tint almost to a black color of both lips and face. An intimate friend and schoolmate (Mr. V. E.) informed me, that at the age of ten years, he was in the habit of visiting his mother's about a mile from town. When he walked about half a square a paroxysm of dyspnœa, palpitation, and lividity would occur. To save time he would be obliged to carry him on his back to the country. He also states that four years ago he visited with him the same neighborhood, and they were four hours walking a mile and a half, so greatly did exercise affect his respiration. I have known Mr. Y. for the last twenty-five years, and believe, that with the exception of cyanosis, his general health has been pretty good ; and although of feeble constitution and general muscular debility, he frequently acted in the capacity of a merchant. I often observed him in a squatting position or sitting upon his heels, with his body bent forwards, a position which gave him some relief in respiration. When perfectly quiet his remissions would extend to hours or days, but the slightest exertion at table would bring on a paroxysm. For the last two years I have observed more emaciation and debility. About six months ago he was taken with symptoms of hectic fever and night sweats, although he continued to go about until three months before death. About this time I was called in and found him laboring under congestion of the lungs, with hectic, attended with dyspepsia and frequent colic ; the latter almost invariably produced by taking the least solid food. There was no diarrhœa. About two months before dissolution he expectorated bloody sputa, which soon subsided under the usual remedies, though there still remained some dyspnœa and cough, with frequent lividity of his lips and face. His pulse was accelerated and irritated, generally regular, though sometimes intermittent ; the night sweats were heavy whether preceded by fever or not ; bowels generally costive, but readily acted upon by aperients or injections. His cough became much more aggravated two weeks before dissolution ; tympanites also occurred, followed by slight œdema of the feet and ankles with ascites. The dyspnœa increased to orthopnœa, and sputa mixed with blood were discharged for four or five days before death, which eventually took place on the 19th of February, 1850.

Autopsy twelve hours after dissolution, assisted by Dr. J. Gregg Gibson, Mr. Wm. A. Waters, and P. Grove.

The emaciation of his frame was very great.

*Thorax.*—The pericardium contained some four or five ounces of serum. The heart rather smaller than ordinary, right auricle and ventricle much larger than the left. The right side of the heart very much distended with blood, particularly the right auricle and coronary veins, the latter were as much so as if filled with a wax injection ; the right auricle was unusually large, and the walls twice as thick as usual; the foramen ovale was closed, but the fossa ovalis and annulus ovalis were located rather higher up than usual, or nearer the descending cava. The right auriculo-ventricular opening was larger than usual. The right ventricle was much larger and thicker than the left ; it was nine lines thick, the columnæ carneæ were also thicker than in the left ventricle, with some fibrinous deposites in the meshes of the chordæ tendineæ. The *Aorta* arose from the *right ventricle* in common with the left ventricle, from the base of the interventricular septum. The aperture in the interventricular septum admitted the passage of the middle finger with great freedom; indeed the opening was three quarters to an inch in diameter between the ventricles. The sigmoid valves of the aorta were normal.

*Pulmonary Artery.*—Here was a most serious malformation. It arose from the right ventricle or interventricular septum, by a

*small orifice* only three or four lines in diameter; the mouth of the artery was encircled with a tendinous band, and that circular band was crossed at the mouth by *another band* just in the *rear* of the circular band, which lessened the diameter of the aperture or mouth of the P. artery about one and a half lines; the larger aperture being the mouth of the pulmonary artery, and the smaller aperture terminating in a small blind sac a few lines in diameter and length, ending in the muscular tissue of the heart. Beyond this contracted mouth of the P. artery the vessel enlarged to its full size, and contained two large semilunar valves in lieu of three. Some fatty deposite was found between the mouth of the pulmonary artery and the valves, and likewise in the blind sac.

The *left auricle* appeared normal in the interior, except that the auriculo-ventricular opening was smaller than natural, barely admitting the little finger.

The *left ventricle* was small and concentrically thickened, the wall about six lines thick, the columnæ carneæ of the mitral valve were smaller than those of the tricuspid.

The aorta arose from the upper and anterior end of the left ventricle in common with the right ventricle, and also anterior to the mitral valve. It looked more into the right than the left ventricle, as the semilunar valves could be more readily seen in the right than the left ventricular cavity.

The lungs were both adherent posteriorly to the pluereæ costales, the lower portion of both were tuberculous, and the upper portions were deeply engorged, and in places hepatized. The bronchial glands were as large as hickory nuts, of a dark venous color. There was also a calcareous deposite at the bifurcation of the right bronchiæ.

*Abdomen.*—Evacuated two gallons of water from the peritoneal sac; surface of the intestines blanched.

Omentum absorbed entirely to the colon; the transverse and descending colon down to the rectum contracted to the size or diameter of the index finger.

Liver studded with tubercles superficially, interior healthy as to structure, but of darker color than usual, as if from venous congestion. Spleen likewise darker than normal. Right kidney healthy, the left engorged slightly in the cortical portion.

